# On the Form of the Skull as Supplying a Distinctive Character of the Varieties of Mankind

**Published:** 1847-10

**Authors:** 


### Art. VIII.

*Ueber Schädel-bildung, zur festeren Begrundung der Menschen-rassen. Von* Prof. Dr. A. Zenne.—*Berlin,* 1846.

*On the Form of the Skull as supplying a Distinctive Character of the Varieties of Mankind.* By Professor Zenne, m.d. Part I.—*Berlin,* 1847.

In the commencement of his work, the author objects to that view of the origin of the human race from one original pair, which is founded on the Old Testament. In the third chapter, entitled "Schädel-polarität," he complains that, on examining the rich collection of skulls in the Berlin Museum, in reference to Retzius's arrangement of the various nations, he, as well as other more experienced anatomists, found considerable difficulty in determining to which of the last-mentioned author's classes several of the specimens belonged. He says, for example, that the skull of a Swede, presented to the Museum by Retzius, was found to be shorter than the skulls of two Russian ladies in the same collection; further, that Retzius refers the Incas to the brachycephalæ, while, in the opinion of our author, their crania are remarkably long, and he appeals to the long Peruvian skull figured in Prichard in proof of the correctness of his criticism. In conclusion, he finds fault with the term "Tartars," as indefinite, and applied by turns to Turks and Mongols.

We must take the liberty, *in limine,* to point to the great difficulties connected with the formation of ethnographic collections of crania, and to the wide difference between these and collections of other objects in natural history. The race of man includes but one species; the different types are but varieties, presenting only differences in degree, together with hybrids, produced by crosses of such varieties. More than one national type is indigenous in most countries. In a large proportion of countries great migratory movements have taken place, and often the remnant of a former race has adopted the language of the more powerful. This appears to have been the case in North Germany, where the people, originally Sclavonic, have in process of time adopted both the language and the customs of the Germans, and thereby become amalgamated with the German race. Similar phenomena obtain in many parts both of the old and new world. Moreover, we must not overlook the influence of smaller immigrations of foreign nations, as well as of the considerable number of individual peculiarities of form, which present themselves in highly civilized countries.

Under these circumstances very extensive investigations are necessary to the determination of primitive forms, and they should be made as far as possible both on living individuals and on prepared crania; not forgetting that such investigations require an eye accustomed to the examination of objects of natural history. Rightly to study the forms of crania, we ought, if possible, to have a large number of specimens before us, which must be examined in different positions, and all in the same position at the same time. As arranged in museums, the face is generally the only part visible. To make a more complete examination costs a great deal of trouble, and easily puts a whole collection into disorder, for which reason such can seldom be made in a large museum without damage; and yet, by an examination of one or more specimens only in the usual manner, no certain opinion can be formed, unless the observer has previously acquired very considerable experience.

In the great museums of Paris, Berlin, and London, the number of national skulls is very great, and, if we do not mistake, a large number of them have been collected on the battle-fields of 1812-1814; a still larger number have been contributed by travellers, or purchased of dealers in objects of natural history; *but the necessary ethnological information is wanting with respect to the majority.* As we have already remarked, it is not sufficient to know that a skull is that of a Frenchman, Englishman, Russian, and so forth, for France is peopled by Basques, Germans, Normans, several Celtic varieties, &c.; the case in England is much the same, and the learned author knows well, that the number of different races is still greater in Russia, that vast empire, to which most European nations are thought to owe their origin. Thus, Count Demidoff reckons fourteen different races as belonging to the Crimea alone. We cannot, therefore, attach any importance to the remark, that two Russian crania were found to be longer than a Swedish; nor should it be forgotten that Retzius does not use the term Russian, but Sclavonic, and our author evidently agrees with him touching the form of the head of these latter when he says, pp. 12: "Der weit verbreitete Stamen der Slawen welche vorherrschend Turanische Schädelbildung haben;" and p. 20, where he speaks of the Turanian race as synonymous with the Mongolian, in his class "Breitschädel," while, on the other hand, he places his Iranian or Caucasian race in the class of "Hochschädel."

Our author's objection to placing the Incas among the brachycephalæ is quite as much a mistake, the more remarkable as he refers to Prichard's 'Researches into the Physical History of Mankind,' vol. i. plate I. The skull there figured, from the valley of Titicaca in Peru, which, together with a number of similar specimens, was brought to Europe by Pentland, and deposited in the Hunterian Museum, is not asserted by Prichard to be that of an Inca. As far as we know, all ethnographers consider these long crania as having belonged to a people inhabiting Peru before the advent of the race of Incas. Tschudi, who found families of these long-headed Peruvians still living in the department of Junin, and who, like Pentland, examined a number of ancient tombs of this race, calls these aborigines, Huauca. We imagine that the best authority on this subject is Morton, whose classical work 'Crania Americana,' Philadelphia, 1839, our author quotes on two occasions, and who says of the Incas, p. 115,—"the skull in this people is remarkable for its small size, and also, as just observed, for its quadrangular form. The occiput is greatly compressed, sometimes absolutely vertical; the sides are swelled out, and the forehead is somewhat elevated, but very retreating." We think it quite unnecessary to add any reasons of our own in justification of the arrangement complained of.

Prof. Zenne considers the appellation Tartars inexact, being applied sometimes to Turks, sometimes to Mongols; and to Tungusians, he might have added, among whom the Maudschu Tartars are also included. The term Tartar is certainly not a very exact one, no more is Tartary, but on the other hand, the name is so remarkable in history, and so generally in use, that we think it possesses a just claim to a place in the list of nations, especially as we feel convinced that all the races designated by the name, are brachycephalic. He also disapproves of Retzius's arrangement, as being founded on a simple polarity or antithesis of cranial forms, contending that the cavity of the skull must be measured in a threefold manner—namely in relation to its length, breadth, and depth. We have only to reply that this arrangement is based upon extensive and laborious researches, and seems to us all the more likely to be correct, as it is found to be intimately connected with the development of the cerebral lobes; and we may add, that at the Christiania meeting of Savans in 1844, Retzius mentioned it as his opinion, that several transition classes between those already determined in his scheme, would be necessary, but that their formation would require the most accurate and probably extensive examinations. For example, the people called by Dieffenbach, (Travels in New Zealand, London, 1843,) "True Polynesians," and considered by him as a branch of the Malay stem, have almost square skulls, not short but high, with large tubera parietalia. They form a transition from brachycephalæ to dolichocephalæ, but are most nearly allied to the first named.

It is doubtless within the bounds of possibility, to make a really good arrangement of cranial forms, by including in the investigation the three proposed measurements of the cavity, provided always one goes the right way to work, which our author would scarcely seem to have done, when he confounds the face with the skull proper. Thus he says that one recognises "die Formen der Hochschädel" by the oval form of the face of south and west Europeans, as well as of the inhabitants of south and west Asia as far as India; but it is well-known that this oval face belongs as much to the long-oval as to the short-square and round skulls. It may be all very well to flatter one's self on the possession of a high skull like the Apollo Belvidere, but truth obliges us to confess that the skulls of the German, Dutch, Norman, Scandinavian, Celtic, and several other west and south European, as well as west and south Asiatic nations, are long-oval, not high, nay, often even very low, though this circumstance does not by any means prevent the face from presenting a handsome oval. Anatomy teaches us that only a small portion of the skull or brain-pan enters into the composition of the face, forming the forehead; all beneath the eyebrows belongs to the face and jaws, not to the brain-case. If the anterior lobes of the brain are well developed, the forehead is high, and if the face be of a regular oval, the individual may present in front, the characters of our author's " Hochschädel," while in fact he has a long, low, skull. The same erroneous views present themselves with regard to the "Langschädel" concerning which, he says, " endlich in der Form der in den *Kiefern* verlängerten, und im *Gebiss* nach vorn gerichteten Schädel der neger, erschauet man die '*schnauzenartigen Formen der Langschädel.*'" Here the face and brain-pan are confounded in a striking manner.

Our author considers Papuas and Alfourous, as mixed races of Turanians and Sudanians, as also Hottentots and Bushmen of Sudanians and Malays. With respect to the first named, the forms of their skulls are different, and the proposition is besides founded on very uncertain grounds; and as to the latter, it is all but demonstrated that the Hottentots are the aborigines of south Africa, and that the Caffres have at a later period taken possession of their country.

Our author assumes a polarity or antithesis in height and flatness of of the cranium, between the Northern and Southern, as well as a similar contrast between the inhabitants of the Eastern and Western hemispheres; principally shown in the presence of an interparietal bone among the inhabitants of the New World. He likewise asserts that the straight facial line is paramount in the North, while prominent cheek bones characterize the inhabitants of the South. But the so-called " True Polynesians" have high skulls, while, on the contrary, Scandinavians have in general long and low ones; flat heads, the lowest of all cranial forms, are found in Oregon, while the Araucanians of Chili have high skulls. The second assumption is equally far from the truth, for Greenlanders have large and prominent cheek bones, and, according to Meyen, the Tagalians of the South Sea, and, according to Dieffenbach, several Polynesian nations have a form of face resembling that of Europeans. (" In their features they approach the Caucasians"—" On true Polynesians." p. 4.) One thing at least we may be sure of, that their cheek bones are not more prominent than those of Greenlanders.

The interparietal bone, which Zenne thinks characteristic of Americans, has been as yet only observed among Peruvians in the New World, and he allows that, though rarely, it does now and then occur among Europeans. Our opinion is, that this bone will occasionally be found in the skulls of all the varieties of man, certainly quite as often in all other as in European.

That the change in the form of the skull brought about by artificial means, among several American tribes, is connected with an observed natural tendency towards the form so much admired by them, we are quite prepared to admit.

Zenne considers that the highest portions of the globe were first inhabited; namely in the east, Iran, Turan, and Sudan; in the west, the Bolivian, Guianian, and Appalachian chains. He places the high-skulled race in the north, the broad-skulled in the middle portions, and the long-skulled in the South, according to the following scheme:

NORTH.

WEST. | EAST.

| Western Hemisphere, or New World. | Eastern Hemisphere, or Old World. |
|---|---|
| I. *High Skulls.* | |
| 4<br>Appalachian, or<br>Natches Race. | 1<br>Caucasian, or<br>Iranian Race. |
| II. *Broad Skulls.* | |
| 5<br>Guianian, or<br>Carib Race. | 2<br>Mongolian, or<br>Turanian Race. |
| III. *Long Skulls.* | |
| 6<br>Peruvian, or<br>Inca Race. | 3<br>Ethiopic, or<br>Sudanian Race. |

SOUTH.

That the above arrangement is not a happy one, is evident from the remarks we have already made ; we will only add, that Zenne places his high skulls, the Iranian and Natches races, farthest north. In the Old World, at least, the Turanian ("broad-skulls" of Zenne), including Lapps, Samoiedes, Yakutans, and Kamschatkans, is the recognised Northern race. The Flatheads of Oregon already mentioned (very low), are found further north than the Natches, who, emigrating from Anahuac, have in later times dwelt in Florida and Louisiana. Still further north do we find races with skulls, differing, it is true, in form, but yet not high ones, namely, Esquimaux, Hurons, &c.

To place Guianians or Caribs in the same class with Mongols is also an evident mistake, as the former have long, the latter short skulls.

According to our author, "long skulls" are represented in the Eastern hemisphere by the Negro race, while in the Western, Incas (Tschudis "Huancas") present the typical cranial forms of the inhabitants of the South ; but the Malays and Papuans of the South Seas have *not* long skulls, and must be placed in the author's class "Breitschädel ;" and he seems to have overlooked the fact, that the Puelches and Araucanians, the former in the Argentine Republic, the latter in Chili, do neither of them belong to his "Langschädel," but, like the Malays, have a Mongolian form of skull.

At page 22 we find the diagnoses of our author's six races of men, but he does not mention what nations are included in each race ; had he subjected the correctness of his system to this trial, he would doubtless have himself observed the very same objections to it, which we have pointed out.

The plates are good and true to nature—no slight praise,—and just on that account are they invaluable witnesses to the truth of our criticisms.

Those races, both in the Old and New Worlds, which our author refers to the same class, present in these plates nearly opposite forms ; for example, the short, high, flat-necked Natches is placed alongside the long, lower German skull, remarkable for its prominent neck, and the long

Macusian forms a striking contrast with the short high Mongolian cranium. The same thing would have been observed if a *real* Inca skull had been placed by the side of a Negro's [we have already remarked that our author confounds Huancas with Incas.]

Towards the close of the work Retzius is mentioned as having adopted the theory of several primitive races; we beg to remind our readers that he has, in his published works, hitherto only treated of cranial forms, and is of opinion that the subject of races cannot be investigated with a chance of success before these forms have been more exactly determined and arranged, fairly acknowledging that the scheme of races he has proposed is only an attempt at or approximation to a final arrangement.

Although we have been obliged to find considerable fault with our author's production, we feel pleasure in bearing witness to his learning, and his very respectable attempt to improve the yet undeveloped science of ethnographic craniology. Had he paid more attention to details, and possibly been more at home in anatomical investigations, and more accustomed to the examination of objects of natural history, he would certainly have been more successful in his researches, and have earned a greater meed of praise than we have felt justified in bestowing.

